# Occurrence of Functional Molecules in the Flowers of Tea (*Camellia sinensis*) Plants: Evidence for a Second Resource

**DOI:** 10.3390/molecules23040790

**Published:** 2018-03-29

**Authors:** Yiyong Chen, Ying Zhou, Lanting Zeng, Fang Dong, Youying Tu, Ziyin Yang

**Affiliations:** 1 Key Laboratory of South China Agricultural Plant Molecular Analysis and Genetic Improvement & Guangdong Provincial Key Laboratory of Applied Botany, South China Botanical Garden, Chinese Academy of Sciences, Xingke Road 723, Tianhe District, Guangzhou 510650, China; yychen@scbg.ac.cn (Y.C.); yzhou@scbg.ac.cn (Y.Z.); zenglanting@scbg.ac.cn (L.Z.); 2 Tea Research Institute, Guangdong Academy of Agricultural Sciences & Guangdong Provincial Key Laboratory of Tea Plant Resources Innovation and Utilization, Dafeng Road 6, Tianhe District, Guangzhou 510640, China; 3 College of Advanced Agricultural Sciences, University of Chinese Academy of Sciences, No. 19A Yuquan Road, Beijing 100049, China; 4 Guangdong Food and Drug Vocational College, Longdongbei Road 321, Tianhe District, Guangzhou 510520, China; dongfangxyz@163.com; 5 Department of Tea Science, Zhejiang University, 388 Yuhangtang Road, Hangzhou 310058, China; youytu@zju.edu.cn

**Keywords:** amino acid, aroma, *Camellia sinensis*, polysaccharide, saponin, tea flower

## Abstract

Tea (*Camellia sinensis*) is an important crop, and its leaves are used to make the most widely consumed beverage, aside from water. People have been using leaves from tea plants to make teas for a long time. However, less attention has been paid to the flowers of tea plants, which is a waste of an abundant resource. In the past 15 years, researchers have attempted to discover, identify, and evaluate functional molecules from tea flowers, and have made insightful and useful discoveries. Here, we summarize the recent investigations into these functional molecules in tea flowers, including functional molecules similar to those in tea leaves, as well as the preponderant functional molecules in tea flowers. Tea flowers contain representative metabolites similar to those of tea leaves, such as catechins, flavonols, caffeine, and amino acids. The preponderant functional molecules in tea flowers include saponins, polysaccharides, aromatic compounds, spermidine derivatives, and functional proteins. We also review the safety and biological functions of tea flowers. Tea flower extracts are proposed to be of no toxicological concern based on evidence from the evaluation of mutagenicity, and acute and subchronic toxicity in rats. The presence of many functional metabolites in tea flowers indicates that tea flowers possess diverse biological functions, which are mostly related to catechins, polysaccharides, and saponins. Finally, we discuss the potential for, and challenges facing, future applications of tea flowers as a second resource from tea plants.

## 1. Introduction

Tea (*Camellia sinensis*) is an important crop in over 30 countries, including China, Japan, India, and Kenya. Tea leaves are generally used to make the most widely consumed beverage, aside from water. The beverages made from tea leaves possess characteristic tastes and flavors, and have many health benefits, because tea leaves contain polyphenols, caffeine, amino acids, aromatic compounds, vitamins, and carbohydrates [1,2,3]. The most utilized part of the tea plant is the leaves. Thus, less attention has been paid to tea flowers. Since the application of asexual propagation to tea plants, tea flowers have become a “waste resource”, competing with tea leaves for water and nutrients. To promote the yield and quality of tea leaves, some chemicals, such as ethephon and α-naphthalene acetic acid, have been used to suppress tea plant blossoming [4], which generally occurs from September to December. The tea flower yield is 3000–12,000 kg/year/hectare tea plantation. In China, over 4.0 billion kg of tea flowers are available annually. Furthermore, after removing tea flowers, the yield and quality of tea leaves are enhanced by ~30% the next year (http://baike.baidu.com/link?url=kuRD4sm6LdTlTDfdYAMwJQaa_2QPoUi4_Hh8EWySpXiGJax6pFevl1IhtGcjqEqz9ZuShxXFoNeNccPNv47ME_). In the past 15 years, researchers have attempted to discover, identify, and evaluate functional molecules from tea flowers, resulting in a number of insightful and useful discoveries. There is a great deal of research on the flowers of tea plants, most of which is focused on isolation, identification, and analysis of certain families of metabolites or proteins in tea flowers. However, so far, comprehensive evaluations of these functional molecules in tea flowers compared to tea leaves is unavailable. Furthermore, the advantages and disadvantages of tea flowers as a new resource are still unclear. Here, we summarize the recent investigations into these functional molecules in tea flowers, including functional molecules similar to those in tea leaves and the preponderant functional molecules in tea flowers, as well as the safety and biological functions of tea flowers. Finally, we discuss the potential and challenges of future applications of tea flowers as a second resource from tea plants. The information summarized in this review will contribute to the future application of tea flowers.

## 2. Functional Molecules Similar to Those in Tea Leaves

Metabolites are not limited to a sole plant tissue and can be distributed among several tissues. Tea flowers contain a similar chemical composition as tea leaves. Polyphenols, such as catechins and flavonols; methylxanthines, such as caffeine; and amino acids, such as theanine, are representative metabolites in tea leaves [3]. All of these metabolites also occur in tea flowers.

### 2.1. Catechins

**Figure 1:**
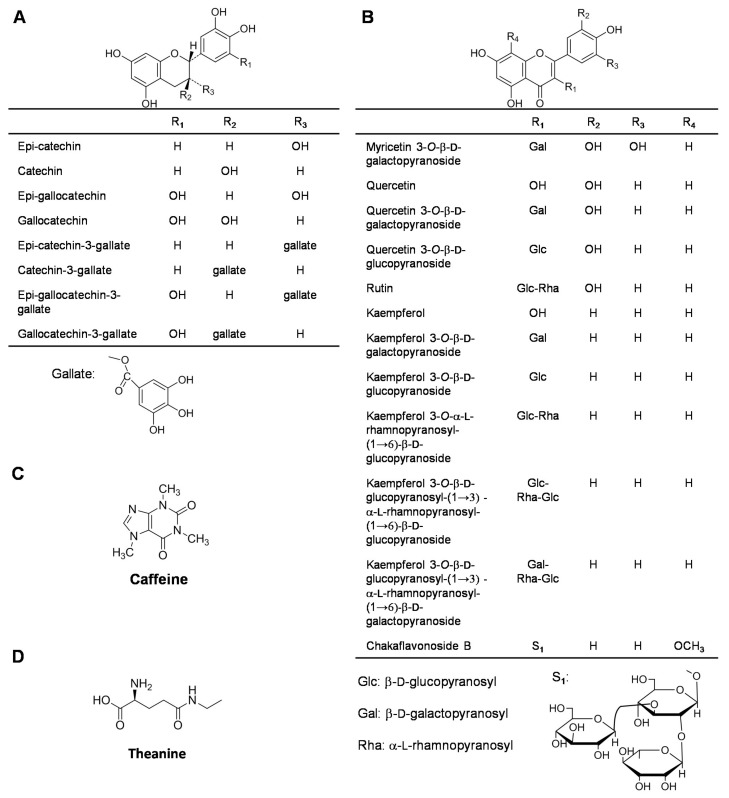
Chemical structures of catechins (**A**), flavonols (**B**), caffeine (**C**), and theanine (**D**) identified in tea flowers. These compounds are representative metabolites in tea leaves. References: [4,8,9,15].

Catechins are the main polyphenols in tea leaves, accounting for 70–80% of polyphenols. The total amount of catechins is generally more than 12% in tea leaves (dry weight) [3,5]. The catechins identified in tea leaves include monomeric catechins, polymeric catechins, proanthocyanidins, condensed tannins, and catechin derivatives, such as methyl catechins and glycosidic catechins [6]. Catechins play important roles in the quality of the tea leaves. Moreover, numerous in vitro and in vivo studies report the beneficial health-related properties of tea leaves and their catechins [1,2]. Therefore, the occurrence of catechins in tea flowers is of great interest. Eight monomeric catechins, catechin, epicatechin, gallocatechin, epigallocatechin (EGC), gallocatechin gallate, epigallocatechin gallate (EGCG), catechin gallate, and epicatechin gallate, have been detected in tea flowers (Figure 1A) [4,7,8,9]. Although the contents and components of catechins vary among different regions or cultivars of tea flowers, EGCG, epicatechin gallate, and EGC have been identified as the main catechins in most tea flowers from different regions or cultivars [4,9]. These three catechins also account for most of the monomeric catechins in tea leaves [3,6,10]. In contrast to tea leaves, tea flowers contain relatively lower contents of catechins (Table 1). The catechin contents increase after budding, reaching their maximum values when the petals started to split and then decreasing to their minimum values at the full-bloom stage [11,12]. Moreover, different tea floral organs contain different components. For example, compared with other floral organs, the calyx contains the highest amount of EGCG [11].

### 2.2. Flavonols

Flavonols are important components for the color of green tea infusions and also contribute to the antioxidant capabilities of tea leaves [3]. Flavonols predominantly occur in their glycosidic forms rather than in their non-glycosylated forms (aglycones). The aglycones of the main flavonols in tea leaves are quercetin, kaempferol, and myricetin. The sugar moieties consist of glucose, rhamnose, galactose, arabinose, and fructose. Mono-, di-, and tri-glycosides of flavonols have been identified in tea leaves [13]. Flavonols (including flavonol glycosides) account for 2.0–3.0% of the tea leaf dry weight [14]. To date, 12 flavonols have been isolated and identified in tea flowers (Figure 1B) [8,9,15]. Like tea leaves, tea flowers contain glycosides of kaempferol, quercetin, and myricetin [8]. Recently, a new flavonol glycoside, chakaflavonoside, was identified in tea flowers [9]. The total flavonol contents accounts for 0.5–1.2% of the tea leaf dry weight [9].

### 2.3. Caffeine

Caffeine is a representative metabolite in tea leaves. Among the plant species that contain caffeine, tea leaves have relatively high amounts, accounting for ~2.0–3.0% of the tea leaf dry weight [16,17]. In contrast to tea leaves, tea flowers contain less caffeine, accounting for ~0.3–1.1% of the tea flower dry weight (Figure 1C) [4,9]. Caffeine present in tea flowers is synthesized in the tea flowers, not transported from the tea leaves [6,18]. Caffeine has several occasional side effects, including palpitations, gastrointestinal disturbances, anxiety, tremor, increased blood pressure, and insomnia, which have resulted in an increasing demand for decaffeinated tea [19,20]. Supercritical fluid extraction with carbon dioxide can be employed in the decaffeination process, which avoids introducing toxic residues from extraction solvents. However, this extraction technique is expensive, and flavors and aromas may be lost during the extraction process [21]. Because of the presence of catechins and the low level of caffeine in tea flowers, it has been proposed that tea flowers may be used to make an alternative tea beverage [4]. 

### 2.4. Amino Acids

There is a relationship between the quality of green tea and its amino acid content [22,23]. Aromatic amino acids are important precursors of aromatic compounds in tea leaves and contribute to the aromatic quality of tea leaves [3]. In addition, theanine, a major amino acid in tea leaves, also has many biological effects, such as a relaxation effect in humans and cooperative effects with anti-tumor agents against cancer [24]. In total, 26 amino acids have been detected in tea leaves. The total content of free amino acids accounts for 1.0–4.0% of the tea leaf dry weight, and theanine accounts for 1.0–2.0% of the tea leaf dry weight [3,6]. In tea flowers, theanine is also the most abundant free amino acid, and the total content of free amino acids accounts for 0.8% of the tea flower dry weight (Figure 1D) [25]. Moreover, the contents of some aromatic amino acids, such as L-phenylalanine, decrease during floral development and mostly accumulate in the anther of tea flowers [26].

## 3. Preponderant Functional Molecules in Tea Flowers

Tea flowers contain representative metabolites similar to those of tea leaves, such as catechins, flavonols, caffeine, and amino acids. However, the concentrations and compositions of these metabolites in tea flowers did not show a preponderance over those in tea leaves. Thus, it was of interest to discover the preponderant functional molecules in tea flowers. 

### 3.1. Saponins

**Figure 2:**
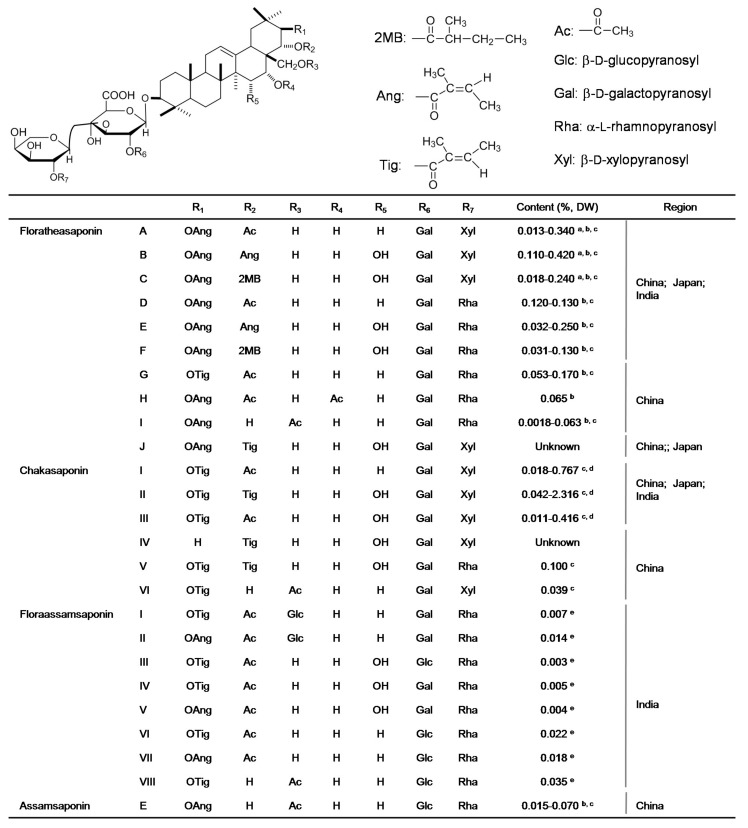
Chemical structures, contents, and regions of saponins identified in tea flowers. The figure was modified and reproduced based on review by Matsuda et al., 2016 [33]. DW, dry weight of tea flowers. ^a^ Ref: [15]; ^b^ Ref: [27]; ^c^ Ref: [28]; ^d^ Ref: [34]; ^e^ Ref: [35].

More than 100 saponins have been isolated and identified from different tissues of tea plants, and most categories of saponins are found in tea seeds [6]. To date, 25 saponins have been found in the tea flowers cultivated in China, Japan, and India (Figure 2) [15,27,28,29,30,31,32,33,34,35]. In contrast to tea leaves (0.04–0.07% of tea leaf dry weight) [36], tea flowers contain greater levels of saponins (0.47–4.23% of tea flower dry weight) [34]. Different regions/cultivars of tea flowers have significant effects on the contents and components of saponins. Tea flowers from western China contain high levels of chakasaponins I–III, while tea flowers from eastern China and Japan contain high levels of floratheasaponins A–F [33,34]. Furthermore, the floratheasaponin content in Japanese tea flowers varies markedly during the blooming periods and peaks at half bloom [37]. Recently, Matsuda et al. summarized that saponins of tea flowers show multiple biological functions, including antihyperlipidemic and antihyperglycemic effects, gastromucosal protection, antiallergic effects in vitro, antiobesity effects, effects on gastric emptying in mice, and the acceleration of gastrointestinal transit [33].

### 3.2. Polysaccharides

Saccharides make up 20–25% of the total tea leaf dry weight [3]. Tea flowers have equivalent amounts of total saccharides (20–30% of total dry weight) [38]. Among the saccharides in teas, polysaccharides are the most representative bioactive component. Tea polysaccharides are soluble glycoconjugates in which a protein carries one or more carbohydrate chains covalently attached to a polypeptide backbone, usually through *N-* or *O-*linkages [39]. To date, 28 kinds of polysaccharides have been reported in tea leaves, including those of green, oolong, and black teas (Reviewed by Jiang and Xiao, 2013) [40]. In tea leaves, the polysaccharides mainly contain rhamnose, arabinose, glucose, and galactose, and contained little xylose or mannose. In the last five years, the polysaccharides of tea flowers (TFPS) have attracted great interest. In contrast to microwave-assisted and ultrasound-assisted water extractions, a traditional water extraction was proposed as the optimal method for extracting TFPS. This resulted in the greatest TFPS yield and the highest neutral and acid saccharide level in the TFPS [41]. Moreover, enzyme-assisted extraction was able to further increase the TFPS yield and easily enhanced the arabinose, galactose, and galacturonic acid contents [42]. In total, 13 kinds of crude and partially purified TFPS have been identified in tea flowers (Table 2). In general, the molecular weights of the TFPS were greater than those of the polysaccharides of tea leaves, and the molecular weight distribution of the TFPS was wide [42,43]. TFPS contain acid polysaccharides, which are made up of rhamnose, arabinose, galactose, glucose, xylose, mannose, galacturonic acid, and glucuronic acid. The molecular weights and monosaccharide compositions of TFPS were influenced by the extraction methods and tea cultivars. 

### 3.3. Aromatic Compounds

**Figure 3:**
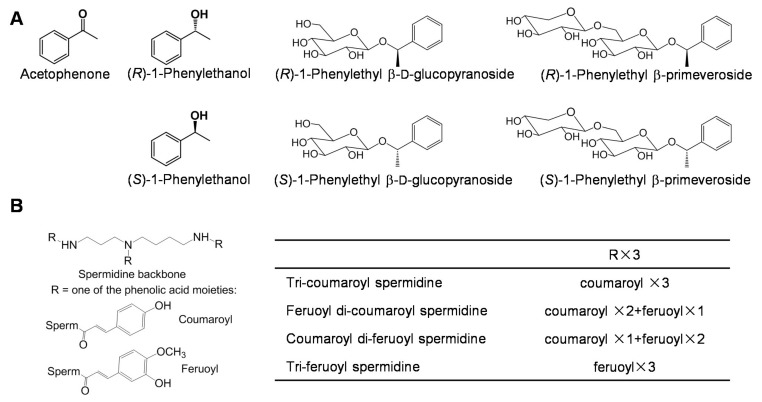
Chemical structures of free forms and glycosidically conjugated forms of aroma compounds (**A**) and spermidine derivatives (**B**) identified in tea flowers. These compounds accumulated in tea flowers with high amounts. References: [26,53,54,56].

More than 600 volatile compounds have been identified in the products of tea leaves. Indeed, most aromatic compounds are produced or enhanced during tea leaf processing. Fresh tea leaves contain relatively fewer aromatic compounds, which are derived from either the terpenoid and shikimate pathways, or by the oxidation of fatty acids and carotenoids (reviewed by Yang et al., 2013) [49]. Aromatic compounds occur in tea leaves not only as free forms, but also as glycosidically bound forms. Because glycosylated aromatic compounds are more water soluble and less reactive than their free aglycone counterparts, many aromatic compounds occur as glycosidic precursors in plant cells, and they are more easily stored in this form [50]. Tea flowers contain a similar composition of aromatic compounds as in tea leaves [12,26,51,52]. Furthermore, two aromatic compounds, acetophenone and 1-phenylethanol, are highly abundant in tea flowers, while they are not highly abundant in tea leaves (Figure 3A) [26,52,53,54]. The levels of the two aromatic compounds increased during floral development and mostly occurred in the anther [26]. Acetophenone is a precursor of aromatic compounds, with aromatic properties that are similar to those of almond, cherry, jasmine, and strawberry. Because of its mild floral odor, 1-phenylethanol is used widely as a fragrance in the cosmetic industry [55]. Both aromatic compounds occur mostly in *Camellia* flowers. However, tea (*C. sinensis*) flowers contain much greater amounts of the two aromatic compounds than other *Camellia* flowers, such as *Camellia sasanqua* [26]. Furthermore, the four glycosidically conjugated 1-phenylethanol, (*R*)-1-phenylethyl β-d-glucopyranoside, (*S*)-1-phenylethyl β-d-glucopyranoside, (*R*)-1-phenylethyl β-primeveroside, and (*S*)-1-phenylethyl β-primeveroside, have been isolated and identified in tea flowers, which provides evidence for the occurrence of glycosidically conjugated 1-phenylethanol in plants for the first time (Figure 3A) [54].

### 3.4. Spermidine Derivatives

A principal component analysis of metabolites (*m*/*z* 70–1000) using ultra-performance liquid chromatography coupled to time-of-flight mass spectrometry demonstrated that significantly different metabolic profiles exist between the metabolic profiles of leaves and flowers at different stages because some characteristic compounds, such as spermidine derivatives, may occur only in tea flowers [56]. Four spermidine derivatives, *N*^1^, *N*^5^, *N*^10^-tri-coumaroyl spermidine, feruloyl di-coumaroyl spermidine, coumaroyl di-feruloyl spermidine, and tri-feruloyl spermidine, have been isolated and identified in tea flowers, while they have not been detected in tea leaves (Figure 3B). Moreover, the spermidine conjugate levels were reduced during the floral stages and were mainly present in the anthers of tea flowers [56]. The content of tri-coumaroyl spermidine, a major spermidine derivative in tea flowers, ranged from 92 μg/g to 181 μg/g (fresh weight), which was more than in most flowers. Spermidine–phenolic acid conjugates are a widely distributed group of plant secondary metabolites that accumulate in the floral parts and have diverse functions in plants, including defense responses against wounding, pathogens, and insects, floral induction, flower formation, sexual differentiation, tuberization, cell division, and cytomorphogenesis (reviewed by Facchini et al., 2002) [57]. In addition, *N*^1^, *N*^5^, *N*^10^-tri-coumaroyl spermidine appreciably inhibits HIV-1 protease [58].

### 3.5. Functional Proteins

Many functional metabolites have been isolated and identified in tea flowers. In contrast, very little attention has been paid to the functional proteins in tea flowers. The protein content accounts for 20–30% of the tea leaf dry weight and 30–50% of the tea flower dry weight [38]. Superoxide dismutase (SOD) is a well-known antioxidant enzyme. Because the use of SOD from animals carries the risk of viral cross-infection, plant-derived SOD has attracted increasing interest. Tea flowers contain ~6000 unit (U) SOD/g dry weight [38], which is higher than that of tea leaves (900–1500 U SOD/g dry weight) [59] and maize kernels and leaves (~4000 U SOD/g dry weight). However, the amount of SOD per mg of protein from tea flowers (2–5 U SOD/mg protein) [38] was much lower than in tea leaves (26–30 U SOD/mg protein) [60] and maize kernels and leaves (45 and 90 U SOD/mg protein, respectively). Moreover, because ingested SOD is degraded into amino acids before being absorbed, there is no indication that the ingestion of SOD or SOD-rich foods has any physiological effects. 

Proteases are considered the most significant industrial enzymes [61]. Because they are active over a wide range of temperature and pH, plant-derived proteases have attracted attention in the fields of biotechnology and medicine. Papain, bromelain, and ficin are extracted from *Carica papaya*, *Ananas comosus*, and *Ficus carica*, respectively, and they are the most widely utilized plant-derived proteases [62]. Proteases, which include plant-derived proteases, such as papain and bromelain [63], and microorganism-derived proteases, such as from *Aspergillus* and *Bacillus* [63,64,65], are generally employed to increase the amino acids in tea infusion (beverage made by tea leaves). However, these proteases did not show potent proteolytic abilities towards proteins from tea infusion, in which the amounts of total amino acids were increased by 0–67%. This may be because these proteases have a weak substrate selectivity towards proteins from tea infusion. Recently, tea flowers were found to contain proteases with hydrolytic abilities against proteins of tea infusion to produce free amino acids. Proteases from tea flowers increased the total amino acids content of tea infusion by 177%, which was greater than the capabilities of commercial proteases [66].

## 4. Safety and Diverse Biological Functions

### 4.1. Evaluation of Mutagenicity, and Acute and Subchronic Toxicity in Rats

The safety of the major functional compounds, such as polyphenols, caffeine, and saponins, present in tea and other common foods has been assessed and confirmed [67,68,69,70]. The safety of tea flowers can be evaluated based on their long history of consumption in China, and toxicity studies on the major functional compounds from tea leaves, which also occur in tea flowers. However, there is limited direct evidence from systematic evaluations of the toxicity of tea flowers. Li et al. reported on the mutagenicity, and acute and subchronic toxic effects of tea flowers in rats [71]. The Ames test showed that tea flower extract (up to 5.0 mg/plate) had no mutagenic effects on the four tested rat strains (TA97, TA98, TA100, and TA102) in the presence or absence of metabolic activation. Tests of the acute toxicity showed that all of the rats were active and normal and had increased in weight. The LD_50_ value was greater than 12.0 g/kg body weight. In their study on subchronic toxicity, the tea flower extract had no dose-related effects on survival, growth, hematology, blood chemistry, organ weights, or pathological lesions. Thus, the tea flower extracts did not possess mutagenic potential and had very low acute and subchronic toxicity towards animals. The no-observed-adverse-effect level for tea flower extract was determined to be 4.0 g/kg body weight/day for rats under the study conditions [71]. The acceptable daily intake was calculated to be ~40 mg (tea flower extract)/kg body weight/day/lifetime. For 70 kg adults, the acceptable daily intake is 2.8 g of tea flower extract, or 9.9 g of dry tea flowers, which are unlikely amounts to be consumed in daily life. Based on these results, tea flower extracts are proposed to be of no toxicological concern [72].

### 4.2. Biological Functions 

The presence of many functional metabolites in tea flowers indicates that tea flowers possess diverse biological functions, which are mostly related to catechins, polysaccharides, and saponins. Direct evidence for these functions has been obtained from tea flower extracts and metabolites isolated from tea flowers. However, previous studies have mostly focused on evaluation of multiple biological functions of extracts of tea flowers and their molecules, and the detailed action mechanisms are still unknown. 

#### 4.2.1. Catechin- and Polysaccharide-Derived Antioxidant Abilities

Antioxidant abilities of extracts of tea flowers were evaluated by in vitro systems using hydroxyl radical and 2,2-diphenyl-1-picrylhydrazyl (DPPH) radical. Ethanol extract of tea flowers showed strong direct scavenging abilities against hydroxyl radical (IC_50_ = 19.7 μg/mL) and against DPPH radical (IC_50_ = 47.6 μg/mL) [7,8]. By isolation and purification, catechins, especially EGCG and EGC, contributed the most to the DPPH radical scavenging ability of tea flowers, which was due to the fact that EGCG and EGC occurred in tea flowers in high amounts, and their gallate moiety structural features may provide more interaction sites with radicals [8]. In in vivo cell systems, the ethanol extract of tea flowers (50 μg/mL) had nitric oxide-suppressing effects in lipopolysaccharide-induced RAW 264.7 cells (derived from murine macrophages), which was proposed to have resulted from occurrence of catechins in ethanol extract of tea flowers [4]. 

In addition to catechins, polysaccharides may also be responsible for the antioxidant abilities of tea flowers. The crude TFPS exhibits equivalent antioxidant abilities as the crude polysaccharides of tea leaves (TLPS) [43]. The IC_50_ values of crude TFPS against superoxide anion were 11.37 μg/mL and 18.29 μg/mL, respectively [43]. The IC_50_ values of crude TFPS against hydroxyl radical were 88.32 μg/mL and 102.37 μg/mL, respectively [43]. The IC_50_ values of crude TFPS against DPPH radical were 64.17 μg/mL and 92.27 μg/mL, respectively [43]. The crude TFPS could be fractionated by chromatography to obtain TFPS with low molecular weights. In contrast to the crude TFPS, the purified TFPS with low molecular weights possess stronger scavenging effects of the superoxide, hydroxyl, and DPPH radicals [45,47]. The administration of the purified TFPS with low molecular weights not only protects the liver lipid peroxidation induced by bromobenzene in mice by enhancing SOD activity, but also attenuates the enhancement of the malondialdehyde content in a dose-dependent manner [73]. Thus, the TFPS could be considered as potential antioxidants.

#### 4.2.2. Catechin- and Polysaccharide-Derived Antitumor Abilities

Way et al. compared the beneficial effects of flowers from six *Camellia*, including *C. japonica*, *C. tenuifolia*, *C. oleifera*, two savory *Camellias*, and *C. sinensis* (tea), against human breast cancer MCF-7 cells [74]. The water extract of *C. sinensis* (tea) flowers (50 μg/mL) showed the strongest effect based on both MTT assays and the cleavage analysis of apoptosis-related molecules. When the cells undergoing apoptosis, the caspase-3 substrate PARP and the pro-apoptotic protein Bid were cleaved by extract of tea flowers. Furthermore, the main bioactivities of the tea flowers were attributed to the presence of EGCG and EGC, which were not detected in the other five *Camellia* species [74].

Xu et al. extracted the TFPS and used DEAE-52 cellulose chromatography to obtain three purified TFPS fractions [45]. Two of the purified TFPS fractions showed relatively stronger suppressive activities on the growth of human gastric cancer BGC-823 cells [45]. After 72 h incubation, the inhibition rate of crude TFPS (200 μg/mL) was 59.18%, while the inhibition rates of the two purified TFPS (200 μg/mL) were above 80%. Moreover, Han et al. investigated the inhibition effect of the TFPS, including the tumor inhibition rate, mouse survival rate, and cellular immunity, on sarcoma 180 tumor (S180)-bearing mice [75]. The continuous administration of the TFPS for 10 days inhibited the growth of transplanted S180 cells (inhibitory rate 60.9% at a dosage of 150 mg/kg) and extended mouse survival. Furthermore, TFPS promoted the plasma interleukin-2, interferon-γ levels, improved the T-lymphocyte subsets CD4^+^ and CD4^+^/CD8^+^ percentages, and increased the delayed-type hypersensitivity response and macrophage phagocytosis. These suggest that the antitumor activity of TFPS can be achieved by improving immune response [75].

#### 4.2.3. Polysaccharide- and Catechin-Derived Immunostimulating Abilities

In vivo and in vitro studies suggested that the polysaccharides from tea leaves possess immunostimulating activities [40,76,77]. The immunostimulating activities of the TFPS were evaluated by Han et al. [75]. The inhibition effect of the TFPS (consisting of two kinds of polysaccharides with peak molecular masses of 31 kDa and 4.4 kDa) on S180-bearing mice was evaluated at dosages of 75, 150, and 300 mg/kg. In contrast to control groups, continuous administration of the TFPS for 10 days suppressed the growth of transplanted S180, prolonged mouse survival, promoted the levels of plasma interleukin-2 and interferon-γ, and improved the T-lymphocyte subsets CD4^+^ and CD4^+^/CD8^+^ percentages. In addition, the TFPS significantly enhanced the delayed-type hypersensitivity response and macrophage phagocytosis. Thus, the TFPS appears to enhance host defense responses to tumors owing in part to the immunostimulating activities [75].

The immunostimulating activities of polysaccharides from tea leaves are affected by the total catechins content in the leaf extract [76]. A complex mixture of tannins with polyphenols and polysaccharides suppresses tumor promotion and carcinogenesis in mice and mouse cell lines [78]. Therefore, the polyphenol–polysaccharide complex may be a potential immunostimulator [76]. It would be of interest to investigate the relationship between, and synergic effects of, the TFPS and catechins on the immunostimulating activities. 

#### 4.2.4. Polysaccharide-Derived Antidiabetic Abilities

In vitro and in vivo studies indicate that TFPS possesses a potential antidiabetic ability. The TFPS obtained by traditional water extraction showed strong inhibitory effects on α-glucosidase. However, the TFPS obtained by microwave-assisted water extraction had very low inhibitory effects on α-glucosidase. In contrast, the TFPS obtained from the different extractions had very low inhibitory effects on α-amylase [42]. The different inhibitory abilities against α-glucosidase of the TFPS obtained using different extraction methods may be the result of the neutral polysaccharides content and different molecular weights of TFPS [46]. Han et al. further isolated two water-soluble polysaccharide fractions from tea flower, TFP-1 (15.9 × 104 g/mol) and TFP-2 (1.12 × 104 g/mol), and evaluated their antidiabetic abilities. Both TFP-1 and TFP-2 (0.2–2.0 mg/mL) can suppress α-glucosidase and α-amylase. Furthermore, the continuous administration of TFP-2 for 3 weeks resulted in a significant reduction in blood glucose levels in alloxan-induced diabetic mice [48]. In addition, in alloxan-induced diabetic rats, the TFPS reduced the blood glucose level, enhanced the weight and the growth rate, and decreased SD rat blood glucose level after the second alloxan injection. Thus, the crude TFPS possesses significant hypoglycemic actions in diabetic rats induced by alloxan and could prevent hyperglycemia [79].

#### 4.2.5. Saponin-Derived Antiobesity Abilities

Obesity is a serious health problem worldwide. A methanolic extract from tea flowers (250 mg/kg and 500 mg/kg, po/d) inhibited body weight increases and the weights of visceral fats in high-fat diet-fed mice. Furthermore, the n-butanol-soluble fraction isolated from the methanolic extract of tea flowers and its main constituent, chakasaponin II, suppressed mRNA levels of neuropeptide Y, which is an important regulator of body weight that affects food intake and energy expenditure, in the hypothalamus. Furthermore, chakasaponin II increased the release of serotonin from the isolated ilea of mice in vitro. These findings suggest that the active saponins are able to suppress the appetite signals in the hypothalamus, leading to reduced food intake and body weight gains [31].

#### 4.2.6. Saponin-Derived Antihyperlipidemic and Antihyperglycemic Abilities 

The methanolic extract and its n-butanol-soluble fraction from tea flowers inhibit serum triglyceride elevation in olive oil-treated mice. Furthermore, among the n-butanol-soluble fraction, floratheasaponins A–C inhibit serum triglyceride elevation, and their activities are more potent than those of theasaponins E1 and E2 obtained from tea seeds [15]. Moreover, Matsuda et al. investigated the effects of chakasaponins I–III on plasma triglyceride and glucose levels in olive oil and sucrose-loaded mice. Chakasaponins I–III (50 and 100 mg/kg) significantly suppressed elevations in plasma triglyceride and glucose levels owing in part to the inhibition of gastric emptying [32].

#### 4.2.7. Saponin-Derived Antiallergic Abilities 

β-Hexosaminidase is stored in secretory granules of mast cells and basophils and is a degranulation marker of mast cells. Therefore, it can be employed to evaluate antiallergic compounds in laboratory animals [80]. Floratheasaponins A–F isolated from tea flowers have inhibitory effects on the release of β-hexosaminidase from RBL-2H3 (rat basophilic leukemia) cells. In particular, floratheasaponins B (inhibitory rate 59.8% at 3 μM) and E (inhibitory rate 52.3% at 3 μM) have more potent activities than the two known antiallergic compounds tranilast (inhibitory rate 22.4% at 100 μM) and ketotifen fumarate (inhibitory rate 27.6% at 100 μM) [27].

## 5. Concluding Remarks and Perspectives 

In the past 15 years, tea flowers have attracted an increasing amount of scientific interest. The International Institute of Tea Flower and International Research and Development Center of Tea Flower were established in Japan and China, respectively. Tea flowers, as a wasted and abundant resource, were recognized as a new food source by the Minister of Health of China in 2013. This indicates that tea flowers may have wide applications in the near future. Recently, a variety of health/functional foods and beverages made with tea flowers have been developed in China and Japan. 

**Figure 4:**
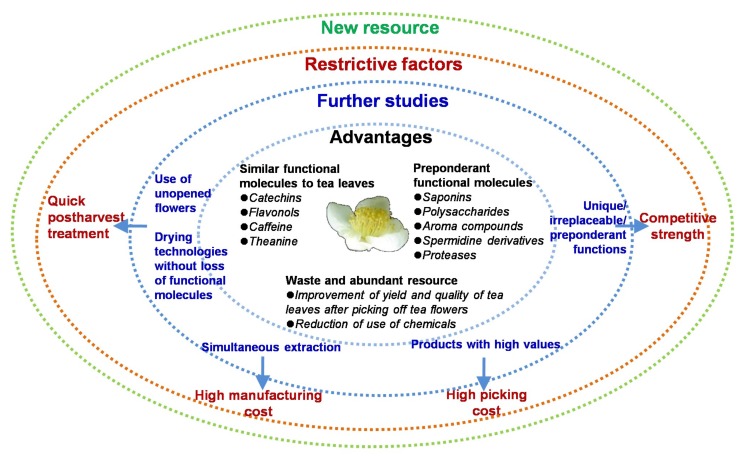
Summaries of advantages, further studies, and restrictive factors of tea flowers as a new resource.

Although many functional molecules occur in tea flowers, the advantages and disadvantages of tea flower extracts and their functional molecules in future applications need to be evaluated. This review provides comprehensive evaluations of the functional molecules in tea flowers compared with those in tea leaves and selects the preponderant functional molecules (Figure 4). In general, low-grade tea leaves are the preferred source for the extraction of functional molecules. In the future, tea flowers could be potential resources for the extraction of functional molecules and could be used as natural value-added products, which would greatly reduce the cost of tea products. However, there are still several factors constraining the future development of tea flower applications (Figure 4), including the following: (1) The high picking cost; in the future, the enhanced quality levels of tea leaf and tea flower products of high values could offset the picking cost. (2) The high manufacturing cost; the establishment of simultaneous extraction protocols and the isolation of multiple functional molecules from tea flowers are needed in the future. (3) Quick postharvest treatment; fresh tea flowers, especially open flowers, have high water content and very active polyphenol oxidases. Thus, they are easily oxidized and turn brown. Most functional molecules have already accumulated at the unopen stage of tea flowers, at which, in contrast to the open stage, flowers can be easily stored for a relatively long time. (4) Competitive strength; in view of the cost and output, it is not possible to utilize all of the functional molecules in tea flowers. Therefore, unique, irreplaceable, or preponderant functions of tea flower extracts and their molecules need to be discovered. In addition, suitable tea flower extract amounts to be consumed in daily life should be evaluated for health benefits for human.

## Figures and Tables

**Table 1:** Comparison of contents of catechins between tea leaves and tea flowers (% dry weight).

Catechins	Tea Leaves ^a^	Tea Flowers ^b^
EGCG	6.81–8.58	0.27–1.97
ECG	1.11–2.07	0.21–0.85
EGC	1.15–1.58	0.03–1.96
EC	0.50–0.69	0.04–0.41
C	0.02–0.11	0.00–0.31

^a^ Reference: [10]; ^b^ References: [4,9].

**Table 2:** The molecular weights and monosaccharide compositions of TFPS.

MW/kDa	Monosaccharide Composition (mol % or Mole Ratios)									References
	Rhamnose	Arabinose	Galactose	Glucose	Xylose	Mannose	Galacturonic Acid	Glucuronic Acid	Fucose	
1.1–483.0	1.7	5.7	10.0	4.8	0.8	1.1	1.2	0.6		[42]
1.1–508.0	3.4	8.7	10.4	4.2	0.8	1.0	9.2	0.5		
1.2–465.0	10.4	22.5	24.9	7.8	1.9	1.6	16.4	0.9		
Unknown	5.0	11.6	11.9	4.3	1.3	2.0	8.4	1.0		[44]
Unknown		14.8	18.1	45.4	12.2	6.9			2.6	[45]
Unknown	11.2	55.2	33.7							
Unknown	21.0	53.3	25.7							
2.6–146	0.4	1.0	1.0	0.4	0.1	0.2	0.7	0.1		[43]
500	1.0	2.9	3.3	1.3		0.5				[46]
167.5	0.8		1.0	1.0	1.2					[47,48]
10.1	2.3	2.3		1.0	0.8					
31.0	Unknown									[41]
4.4	Unknown									

## Abbreviations

DPPH	2,2-Diphenyl-1-picrylhydrazyl
EGC	Epigallocatechin
EGCG	Epigallocatechin gallate
SOD	Superoxide dismutase
TFPS	Polysaccharides of tea flowers
TLPS	Polysaccharides of tea leaves
